# Gone missing or gone crazy? Key statistics about the relation between poor mental health and reports of missing people

**DOI:** 10.1192/j.eurpsy.2025.1625

**Published:** 2025-08-26

**Authors:** G. N. Porfyri, V. Tarantili

**Affiliations:** 1 National and Kapodistrian University of Athens, Athens; 2 General Hospital of Argos, Argos, Greece

## Abstract

**Introduction:**

The definition of a missing individual includes the description of a person whose location is not known alongside alarming signs concerning his state of security and health. Despite thousands of people reportedly missing every year, limited information is available about their identity, the reason of their disappearance and their final evolving.

**Objectives:**

To explore the relation between poor mental health and reports of missing people.

**Methods:**

A review of 29 articles -from 2010 to 2024- on PubMed and Google Scholar regarding people suffering from mental illness who were reported missing.

**Results:**

1 in 5 missing children suffer from mental illness.

1 in 10 missing children is at risk of suicide.

8 in every 10 missing adults suffer from mental health problems.

4 in every 10 people with dementia will go missing eventually, in many cases involuntarily.

3 in 10 missing adults face relationship issues with their partner or relatives.

1 in 50 missing adults struggle with domestic violence.

20% of missing individuals commit suicide, with the majority of them being men who use violent methods such as drowning or hanging.

Those usually reported missing are: females aged 13-17 and men aged 24-30.

Juveniles and adults under 60s usually go missing deliberately, compared to people of different ages.

Reasons for psychiatric patients to go missing, include: disappointment by healthcare professionals; different opinions than their loved ones’ concerning their health; feelings of helplessness; belief that going missing is the only solution.

**Conclusions:**

Mental illness can be both the cause and the consequence of an individual going missing. Missing incidents in many cases represent a veritable moment of crisis and should be approached as such in someone’s life. Unfortunately, when a person is found by authorities, support is rarely offered, potentially strengthening the feelings of hopelessness and despair of the previously missing person.

Mental health care professionals must be in alert after a missing’s person return, ready to offer him their services.

**Disclosure of Interest:**

None Declared

